# Thoracoabdominal flap reconstruction after resection of superficial soft-tissue sarcomas in the chest wall

**DOI:** 10.1093/jscr/rjaa571

**Published:** 2021-01-29

**Authors:** Akio Sakamoto, Takashi Noguchi, Shuichi Matsuda

**Affiliations:** Department of Orthopaedic Surgery, Graduate School of Medicine, Kyoto University, Kyoto, Japan; Department of Orthopaedic Surgery, Graduate School of Medicine, Kyoto University, Kyoto, Japan; Department of Orthopaedic Surgery, Graduate School of Medicine, Kyoto University, Kyoto, Japan

## Abstract

The thoracoabdominal flap is a rotation flap, and is well known for reconstruction of defects following resections for breast cancer, but the flap is not well known for reconstructing defects following resections of soft-tissue sarcomas involving the chest wall. Here we present two patients with superficial chest wall sarcomas consisting of a dermatofibrosarcoma protuberans in a 42-year-old man and a recurrent myxofibrosarcoma in a 76-year-old man. The tumors were resected with the surrounding tissue. The defect was reconstructed with a thoracoabdominal flap elevated from the ipsilateral thorax (medially-based flap). Neither case developed necrosis of the flap or reduced shoulder range of motion. The chest wall presents few options for a donor vessel. The thoracoabdominal flap has an axial blood supply and does not require a microsurgical procedure. A thoracoabdominal flap is a suitable reconstruction option for a defect after the resection of a superficial soft-tissue sarcoma in the chest wall.

## INTRODUCTION

Flap techniques are used to repair soft-tissue defects after resection of malignant soft-tissue tumors [1]. A fasciocutaneous flap is needed when the resection includes the pectoralis major *muscle* and an exposed chest wall. Free vascular flaps of the latissimus dorsi or anterolateral and inferior gluteal thigh are possible; however, they require a microsurgical procedure. The thoracoabdominal flap is a local fasciocutaneous rotation-advancement flap [2, 3].

The thoracoabdominal flap is well known for chest wall reconstruction following breast cancer [2, 3]. Use of the flap for a superficial soft-tissue sarcoma in the chest wall is less well known [4]. In our report of the two cases, we describe the use of a thoracoabdominal flap for reconstruction after resection of superficial chest wall sarcomas consisting of a dermatofibrosarcoma protuberans and a myxofibrosarcoma.

## CASE PRESENTATION

### Case 1

**Figure 1:**
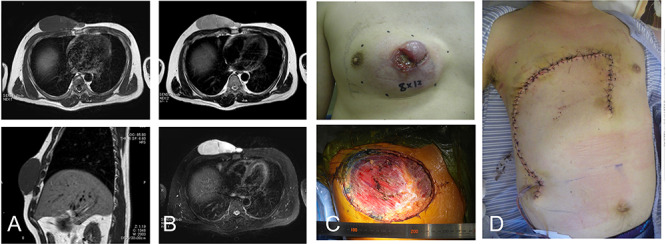
42-year-old male with dermatofibrosarcoma protuberans in the chest wall. Magnetic resonance imaging shows the subcutaneous tumor with low-signal intensity on the T1-weighted image (**A**) and slightly high-signal intensity on the T2-weighted image (B-upper) and high-signal intensity on a T2-weighted fat-suppressed image (B-lower); photograph of ulcerated subcutaneous lesion with subcutaneous extension; after resection of the tumor with the surrounding tissue (**C**), the defect was reconstructed with a thoracoabdominal flap (**D**).

A 42-year-old man presented with a history of a mass on his right chest wall that gradually increased in size over 2–3 years. On physical examination, a protuberant hard, immobile elastic mass that appeared attached to the fascia was palpable on the anterior chest wall. Magnetic resonance imaging (MRI) revealed an 8 × 12-cm subcutaneous tumor. The tumor showed homogenous low-signal intensity on the T1-weighted image and high-signal intensity on the T2-weighted image (Fig. 1A and B). The tumor was attached to the fascia of the pectoralis major *muscle*.

**Figure 2:**
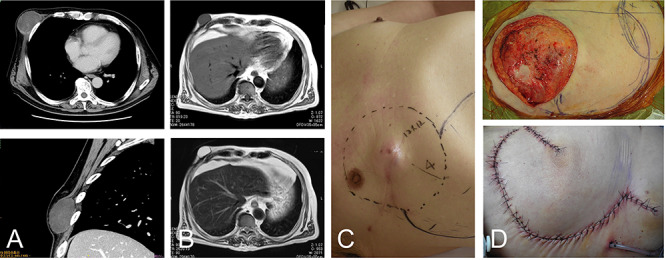
76-year-old male with recurrent myxofibrosarcoma. Computed tomography revealed the initial 6-cm subcutaneous lesion of the patient at 73 years of age (**A**); 3 years after resection of the initial lesion, T1- and T2-weighted images show a 3-cm recurrent lesion with high-signal intensities (**B**-upper) and (B-lower), respectively. Photograph showing protuberant subcutaneous lesion (**C**); defect after resection of the tumor with the surrounding tissue (**D**-upper). The defect was reconstructed with a thoracoabdominal flap (D-lower).

Histological examination of a needle biopsy specimen revealed a dermatofibrosarcoma protuberans. The tumor was resected along with the surrounding skin, subcutaneous tissue, and the underlying pectoralis major *muscle*, leaving a defect of 16 cm in the maximum diameter (Fig. 1C). The defect was reconstructed with a medially-based thoracoabdominal flap (Fig. 1D).

A vertical thoracoabdominal incision of the superficial layer of the skin was made on the lateral side of the defect. During elevation of the rotation flap, the perforators were preserved as much as possible. The lateral side of the flap was rotated medially to the edge of the defect. The patient’s shoulder showed free range of movement. The patient has remained free of tumor over a 4-year postoperative period.

### Case 2

A 73-year-old man presented with complaints of a mass on her right chest wall for several months. Physical examination revealed a 6-cm elastic mass on her anterior chest wall that ranged from soft to hard on palpation. Computed tomography showed a subcutaneous tumor that was 6 cm in diameter (Fig. 2A). Histological examination of a biopsy specimen revealed a myxofibrosarcoma. The tumor was resected with the skin over the tumor, and the wound was closed without reconstruction. After 3 years of the initial operation, the 76-year-old patient underwent MRI, which revealed a recurrent lesion of 3 cm in diameter showing homogenous low-signal intensity on the T1-weighted image and homogenous high-signal intensity on the T2-weighted image (Fig. 2B).

The tumor was resected along with more than 4 cm of surrounding underlying pectoralis major *muscle* and the skin and subcutaneous tissue, resulting in a defect of greater than 12 cm in the maximum diameter. The defect was reconstructed by the same method used in Case 1 for placing a medially-based thoracoabdominal flap (Fig. 2C and D). The patient’s shoulder showed free range of movement. The patient has remained free of tumor over a 4.5-year postoperative period.

## DISCUSSION

A fasciocutaneous flap instead of a skin graft is required for a defect after resection of a chest tumor that includes the pectoralis major *muscle* and also exposes the chest wall. Among the flaps used to repair defects after resection of soft tissue sarcomas, free flaps have a higher rate of complications (43%) than local flaps (17%), and a higher rate of reoperation (13%) than local flaps (4.3%) [5]. Moreover, for a soft tissue sarcoma located on the chest wall, there are not many options for a donor vessel.

The thoracoabdominal flap is a fasciocutaneous rotation flap. In our case series, thoracoabdominal flaps were used for reconstruction after resection of superficial soft-tissue sarcomas in the chest wall. Thoracoabdominal flaps are either medially- or laterally-based flaps [2, 3]. In a comparison with medially-based flaps, laterally-based thoracoabdominal flaps have more stable circulation [6]. A laterally-based thoracoabdominal flap can preserve the lateral intercostal arteries, but is less likely to preserve the superior epigastric perforators [4], while medially-based flap can preserve the superior epigastric perforators. With regard to shoulder range of motion, laterally based flaps used for reconstruction after resection of chest wall cancers may be associated with diminished shoulder range of motion in the short term after surgery [4]. Neither of our two patients, whose defects were reconstructed by medially based thoracoabdominal flaps, developed restricted range of motion involving their ipsilateral shoulder. Therefore, a medially-based thoracoabdominal flap might have merit for preventing reduced shoulder range of motion.

A series of 41 patients who underwent resections of malignant chest wall tumors followed by reconstructions using laterally based thoracoabdominal flaps included 35 cases of breast cancer, and four cases of soft-tissue sarcomas, which included 1 myxofibrosarcoma [4]. Our case series of two included a myxofibrosarcoma and a dermatofibrosarcoma protuberans. Both these sarcomas rather frequently manifest as superficial tumors. Myxofibrosarcoma involving subcutaneous is termed as superficial myxofibrosarcoma [7]. The recurrence rate of superficial myxofibrosarcomas is related to the characteristics of the resected specimen. The recurrence rates of wide, marginal, and incomplete surgical margins have been reported to be 25%, 50% and 67%, respectively [7].

Dermatofibrosarcoma protuberans is a low-grade soft-tissue tumor that occurs in the dermis and subcutaneous tissues and recurs in ~20% of resected patients [8]. For both myxofibrosarcoma and dermatofibrosarcoma protuberans, surgical resection is the main treatment and complete resection with a wide margin is needed to reduce the risk of recurrence. Therefore, a safe and simple reconstruction that uses a thoracoabdominal flap would allow a wide resection for superficial chest wall sarcomas.

The thoracoabdominal flap has an axial blood supply, and the reconstruction procedure is simple to perform and does not require a microsurgical procedure. The thoracoabdominal flap can be an option for reconstruction after resection of a superficial soft-tissue sarcoma in the chest wall.
